# Functional variants in *ADH1B* and *ALDH2* are non-additively associated with all-cause mortality in Japanese population

**DOI:** 10.1038/s41431-019-0518-y

**Published:** 2019-09-26

**Authors:** Saori Sakaue, Masato Akiyama, Makoto Hirata, Koichi Matsuda, Yoshinori Murakami, Michiaki Kubo, Yoichiro Kamatani, Yukinori Okada

**Affiliations:** 1 Laboratory for Statistical Analysis, RIKEN Center for Integrative Medical Sciences, Yokohama, 230-0045 Japan; 2 Department of Statistical Genetics, Osaka University Graduate School of Medicine, Suita, 565-0871 Japan; 3 Department of Allergy and Rheumatology, Graduate School of Medicine, the University of Tokyo, Tokyo, 113-8655 Japan; 4 Department of Ophthalmology, Graduate School of Medical Sciences, Kyushu University, Fukuoka, 812-8582 Japan; 5 Laboratory of Genome Technology, Institute of Medical Science, The University of Tokyo, Tokyo, 108-8639 Japan; 6 Department of Computational Biology and Medical Sciences, Graduate school of Frontier Sciences, The University of Tokyo, Tokyo, 108-8639 Japan; 7 Division of Molecular Pathology, the Institute of Medical Sciences, the University of Tokyo, Tokyo, 108-8639 Japan; 8 RIKEN Center for Integrative Medical Sciences, Yokohama, 230-0045 Japan; 9 Laboratory of Complex Trait Genomics, Department of Computational Biology and Medical Sciences, Graduate School of Frontier Sciences, the University of Tokyo, Tokyo, 108-8639 Japan; 10 Laboratory of Statistical Immunology, Immunology Frontier Research Center (WPI-IFReC), Osaka University, Suita, 565-0871 Japan

**Keywords:** Genetic variation, Clinical genetics, Genetics research, Outcomes research

## Abstract

The functional variants involved in alcohol metabolism, the A allele of rs1229984:A > G in *ADH1B* and the A allele of rs671:G > A in *ALDH2*, are specifically prevalent among East Asian population. They are shown to be under recent positive selection, but the reasons for the selection are unknown. To test whether these positively selected variants have beneficial effects on survival in modern population, we performed the survival analyses using the large-scale Japanese cohort (*n* = 135,974) with genotype and follow-up survival data. The rs671-A allele was significantly associated with the better survival in the additive model (HR for mortality = 0.960, *P* = 1.7 × 10^−5^), and the rs1229984-A had both additive and non-additive effects (HR = 0.962, *P* = 0.0016 and HR = 0.958, *P* = 0.0066, respectively), which was consistent with the positive selection. The favorable effects of these alleles on survival were independent of the habit of alcohol consumption itself. The heterogenous combinatory effect between rs1229984 and rs671 genotype was also observed (HRs for AA genotype at rs671 were 1.03, 0.80, and 0.90 for GG, GA, and AA genotype at rs1229984, respectively), supposedly reflecting the synergistic effects on survival.

## Introduction

Functional variants in *ADH1B* and *ALDH2*, which are specifically prevalent among East Asian population, substantially alter enzymatic activity involved in alcohol metabolism and make the population less tolerant to alcohol consumption [1]. The A allele of rs1229984:A > G (hg19 chr4:g.100239319 A > G; NM_000668.5:c.143 A > G [NP_000659.2:p.(His48Arg)]) causes the rapid oxidation of ethanol to acetaldehyde by ADH1B, which increases an aversive reaction to alcohol, while the A allele of rs671:G > A (hg19 chr12:g.112241766 G > A; NM_000690.3:c.1510 G > A [NP_000681.2:p.(Glu504Lys)]) causes the functional deficiency of ALDH2, which slows the metabolism of acetaldehyde [2]. These alleles are common among East Asians (frequency = 0.738 and 0.255), but are rare or at low frequency in other populations (frequency = 0.047 and 0.0003, respectively [3]). These variants are also known to be highly pleiotropic, and associated with many complex human traits. Rs1229984 is associated with body mass index [4] and pulse pressure [5], while rs671 is associated with diseases such as coronary artery disease [6] and intercranial aneurysm [7], as well as affecting quantitative traits such as body mass index [8], uric acid [9], and triglycerides [10]. Intriguingly, although they are the risk alleles for some of the modern diseases, we have shown that they are under strong recent positive selection among Japanese by analyzing the whole-genome sequencing data [11]. The reasons for their positive selection in East Asians are unknown.

In order to test whether these positively selected variants have beneficial effects on survival, we performed the survival analyses using the large-scale Japanese cohort (*n* = 135,974) with genotype and follow-up survival data. We then performed further statistical analyses to estimate the non-additive and combinatory effects of these variants on survival.

## Materials and methods

Clinical information, genotype, and follow-up survival data were obtained from BioBank Japan [12, 13], which collected DNA and serum samples from ~200,000 participants. We obtained informed consent from all the participants, following the protocols approved by ethics committees of RIKEN Center for Integrative Medical Sciences and the Institute of Medical Sciences, the University of Tokyo. The detailed information of participants is summarized in Supplementary Table 1. Genotyping and Quality control of participants are described elsewhere [14]. We analyzed the concordance of genotyping between the SNP array and the whole-genome sequencing (WGS) data (*n* = 1638). The genotype data in this study is deposited on the Japanese Genotype-phenotype Archive affiliated to the DDBJ (DNA Data Bank of Japan), via National Bioscience Database Center (NBDC), Japan. The data is accessible with the accession IDs hum0014 and JGAS00000000114 at https://ddbj.nig.ac.jp/jga/viewer/view/study/JGAS00000000114.

The survival analyses of rs1229984 and rs671 for all-cause mortality were performed by Cox proportional-hazard models, adjusted for age, sex, the disease status, the habit of cigarette smoking and alcohol consumption, and 10 principal components. Primary analysis was performed by assuming an additive model. Next, to estimate the non-additive allelic effect, we additionally included a dominance term which is one if the genotype is heterozygous and zero otherwise [15]. Finally, to investigate the combinatory effect between rs1229984 and rs671 on survival, we obtained hazard ratios (HR) for each of the combination of genotypes at rs1229984 and rs671. We compared them to the expected HRs by assuming that they should be the products of the two HRs of each allele under the null hypothesis where there are no gene × gene combinatory effects. The deviation in the fold change was obtained by dividing the observed HR by the expected. All the survival analyses were performed using R software, version 3.3.0.

## Results

We first genotyped rs1229984 and rs671, two of the variants under positive selection [11]. As both rs1229984 and rs671 were significantly deviated from the QC threshold of Hardy–Weinberg equilibrium (*P*_HWE_ < 1.0 × 10^–6^), we analyzed the concordance of genotyping between the SNP array and the whole-genome sequencing (WGS) data (*n* = 1638). We confirmed the high concordance between the array-based genotype and sequenced genotype (97.6 and 100%, respectively), indicating that the observed deviation from HWE was not caused by genotyping error but by heterogeneity in allele frequency spectra among the regions of Japan.

We then associated the genotype of these variants with the all-cause mortality (*n* = 135,974) [16]. The median follow-up period was 8.08 years, and the number of deaths during the follow-up was 31,403. We observed that in both of the variants, the alleles which make their carriers less tolerant to alcohol showed significantly favorable effects on survival. When we assume an additive effect, the rs671-A allele was strongly associated with the all-cause mortality (HR = 0.960 [95% Confidence Interval: 0.942–0.978] and *P* = 1.7 × 10^–5^), while the effect of the rs1229984-A was modest (HR = 0.983 [0.965–1.001] and *P* = 0.067, Supplementary Table 2). We note that the effects of these alleles on survival were independent of the habit of alcohol consumption itself, as we regressed out the status of alcohol consumption in constructing the Cox proportional-hazard models.

**Fig. 1:**
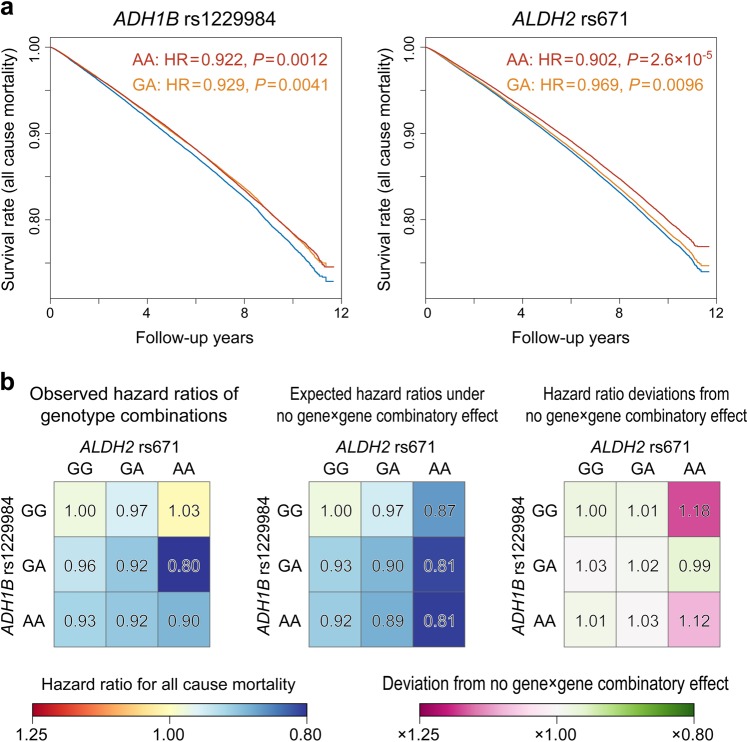
The survival analysis of rs1229984 and rs671 in Japanese population. **a** The standardized and adjusted survival curves for all-cause mortality according to the genotype of rs1229984 and rs671. The blue, orange, and red line indicates the survival curve of participants with the GG, GA and AA genotype, respectively. The GG genotypes were adopted as baselines. (**b**) The observed (left) and expected (middle) hazard ratios for each of the combination of genotypes at rs1229984 and rs671. The expected hazard ratios are calculated by assuming that they should be the products of the two hazard ratios of each allele under the null hypothesis where there are no gene × gene combinatory effects. The deviations of the observed hazard ratio from the expected are shown in fold change (right)

Intriguingly, we found that the association of the heterozygous genotype GA at rs1229984 was comparable to that of the homozygous genotype AA (HR = 0.929 and 0.922) (Fig. 1a and Table 1). The GA and the AA genotype at rs1229984 were both reported to produce 40-fold faster ethanol oxidation by ADH1B than the GG genotype [17]. Thus, in order to clarify the suggested non-linear effect of rs1229984 on survival outcome, we incorporated both the additive and non-additive term into the model [15]. As suggested in the genotype-level association test, we observed a significant additive and non-additive allelic effects of the rs1229984-A on survival (*P*_Additive_ = 0.0016 and *P*_NonAdditive_ = 0.0066), while the rs671-A only had an additive allelic effect (*P*_Additive_ = 3.4 × 10^–5^ and *P*_NonAdditive_ = 0.20, Table 2). Likelihood ratio tests confirmed that the incorporation of non-additive term significantly improved the model fit in rs1229984 (*P*_ANOVA_ = 0.0068), but not in rs671 (*P*_ANOVA_ = 0.20).

**Table 1:** The association of the functional variants within *ADH1B* and *ALDH2* with the all-cause mortality

SNP	Chr	Position (hg19)	Gene	Genotype	Freq.	Hazard ratio (95% CI)	*P-*value
rs1229984	4	100,239,319	*ADH1B*	GG	0.057	Reference	–
				GA	0.354	0.929 (0.884–0.977)	0.0041
				AA	0.589	0.922 (0.879–0.968)	0.0012
rs671	12	112,241,766	*ALDH2*	GG	0.568	Reference	–
				GA	0.367	0.969 (0.946–0.992)	0.0099
				AA	0.065	0.902 (0.860–0.947)	2.6 × 10^–5^

*Freq*. frequency of the genotype

**Table 2:** The additive and non-additive allelic effects of rs1229984 and rs671 on the all-cause mortality

SNP allele	Mode	Hazard ratio (95% CI)	*P*-value
rs1229984 (A)	Additive	0.962 (0.939–0.985)	0.0016
rs1229984 (A)	Non-additive	0.958 (0.929–0.988)	0.0066
rs671 (A)	Additive	0.951 (0.929–0.974)	3.4 × 10^–5^
rs671 (A)	Non-additive	1.020 (0.990–1.051)	0.20

We have previously shown that rs671 and rs1229984 are in trans-chromosomal linkage disequilibrium, reflecting the synergistic natural selection pressure [18]. Thus, we finally tested if there existed gene × gene combinatory effects on survival between rs671 and rs1229984. The combination of the AA genotype at rs671 and the GA genotype at rs1229984 showed the most favorable effect (HR = 0.801 [0.719–0.892] and *P* = 5.5 × 10^–5^, Fig. 1b and Supplementary Table 3). Interestingly, we could observe an upward deviation of the observed HR in the combination of the AA genotype at rs671 and the GG or AA genotype at rs1229984 if we assume that there are no gene × gene combinatory effects (fold change = 1.18 and 1.12, respectively). On the other hand, the incorporation of interactive terms between rs1229984 and rs671 failed to capture the significant effects, probably due to the co-linearity between the terms or lack of statistical power. Taken together, people with the AA genotype at rs671 had the favorable survival outcome than those with the GG or GA genotype as discussed above, and this favorable effect was mostly driven by the subset of people with the GA genotype at rs1229984. By careful examination of the effects of each genotype on survival outcome, we showed a rare example of trans-chromosomal combinatory effects, which we consider to be also essential in analyzing other pleiotropic associations of these loci.

## Discussion

The common functional variants at *ADH1B* and *ALDH2* are specifically prevalent in East Asians, and thus their phenotypic landscape has been understudied. Here we described the association of rs1229984 and rs671 with the all-cause mortality by leveraging the large-scale biobank in Japanese. In both of the variants, the alleles which make their carriers less tolerant to alcohol were shown to have beneficial effects on survival. These observations are consistent with the fact that they are under recent positive selection and in different frequency spectrum among East Asians. They showed beneficial effects on survival even though they are also associated with causing various modern diseases [6, 7]. To have further insights into the model of their effects on survival, we have shown that the rs1229984-A had both the additive and non-additive allelic effect, and that there existed a combinatory effect of rs1229984 and rs671.

One of the alcohol-metabolism related loci, *BRAP-ALDH2*, was also reported to be significantly associated with the length of lifespan in European population in the large-scale study on UK Biobank [19]. The reasons for the positive selection of alcohol-related genes are indecisive, and one of the speculations is that a higher concentration of acetaldehyde was advantageous for parasitic infections endemic in East Asia, past or present [20]. Future functional studies will be awaited to reveal the reasons why these loci have been positively selected in modern Asian populations and hold the survival benefit, and how it is connected with the increasing prevalence of late-onset complex diseases in human populations. Both of the positively selected genes, *ADH1B* and *ALDH2*, are not only substantially explaining the heritability of the alcohol consumption, but also associated with many complex human traits (i.e. pleiotropy) [4–10]. Our results suggested that the favorable effects of the rs1229984-A and rs671-A on survival were independent of alcohol consumption itself, because the associations were conditioned on the habit of alcohol consumption. Their biological mechanism on survival remains elusive, which warrants further studies incorporating the comprehensive phenotypic associations.

In conclusion, the survival analysis on *ADH1B* and *ALDH2* revealed that the functional variants within these loci had favorable effects on survival in Japanese population, and that their non-additive and combinatory allelic effects should be taken into consideration.

## Supplementary information

Supplementary Tables
