# Supplementary material for: Functional variants in ADH1B and ALDH2 are non-additively associated with all-cause mortality in Japanese population
Source: Eur J Hum Genet. 2019 Sep 26;28(3):378–82. doi: 10.1038/s41431-019-0518-y (PMC7028931; doi:10.1038/s41431-019-0518-y)
Supplement: Supplementary file 1 — Supplementary Tables [file 41431_2019_518_MOESM1_ESM.docx]

**Supplementary Tables**

**Functional variants in *ADH1B* and *ALDH2* are non-additively associated with all-cause mortality in Japanese population.**


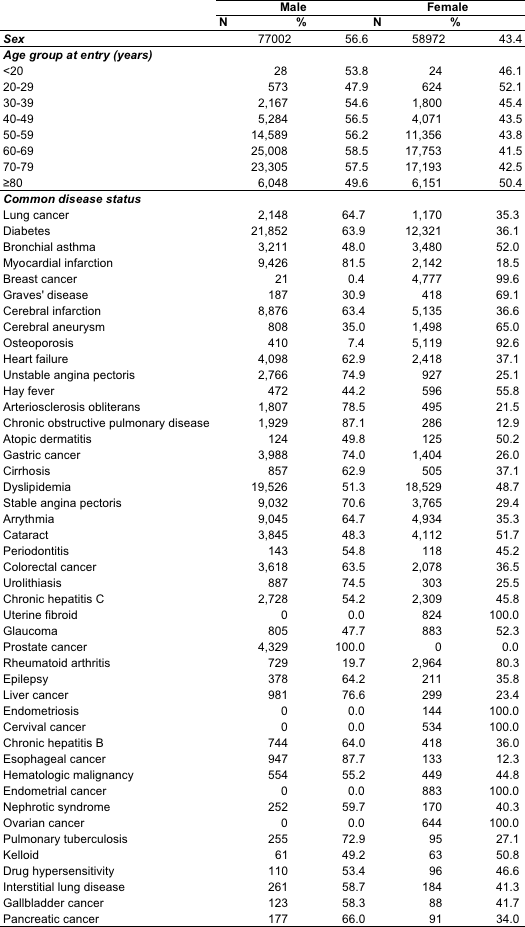


Supplementary Table 1.

Overview of BioBank Japan participants for the survival analysis.

| rsID | A1 | A2 | Freq. | Hazard ratio (95%CI) | *P* |
| --- | --- | --- | --- | --- | --- |
| rs1229984 | G | A | 0.766 | 0.983 (0.965-1.001) | 0.067 |
| rs671 | G | A | 0.248 | 0.960 (0.942-0.978) | 1.7×10^-5^ |

Supplementary Table 2.

The effect of rs1229984 and rs671 on all-cause mortality under additive model.

Freq; Frequency of the A2 allele. Hazard ratios represent the effect of the A2 allele of each variant.

Supplementary Table 3.

The association of each combination of the genotypes at rs1229984 and rs671 with all-cause mortality.

Freq; Frequency of the combination of the alleles.
